# Rarity of Laurence Moon Bardet Biedl Syndrome and its Poor Management in the Pakistani Population

**DOI:** 10.7759/cureus.4114

**Published:** 2019-02-21

**Authors:** Omair A Khan, Ramsha Majeed, Muhammad Saad, Asad Khan, Ayesha Ghassan

**Affiliations:** 1 Internal Medicine, Fauji Foundation Hospital, Islamabad, PAK; 2 Medical Education and Simulation, Fauji Foundation Hospital, Islamabad, PAK

**Keywords:** consanguineous marriage, congenital disorder, laurence moon bardet biedl syndrome

## Abstract

Laurence Moon Bardet Biedl syndrome is characterized as a rare genetic disorder, with a wide range of presenting symptoms such as mental retardation, decreased visual acuity, obesity, hypogonadism, and polydactyly. The diagnosis of this syndrome is easily overlooked due to its rarity, with a prevalence rate of one in 125,000-160,000 reported within Europe. Delayed diagnosis and inappropriate management may lead to an irreversible loss of functions. The most significant of these losses include loss of vision, cardiac problems, and renal abnormalities. These dysfunctions critically impact the mental faculties and personal life of a patient. Our case presented with striking features of this syndrome, but due to a lack of awareness, her family was not adequately counseled. Both the family and the patient were not equipped with the necessary knowledge regarding the nature of her disease and its prognosis. The patient was mismanaged and kept ignorant of the importance of a proper follow-up. This necessitates a multidisciplinary team approach towards such cases so that their disease can be adequately managed. The early diagnosis and symptomatic management of complications as they arise remain the most important and vital step in the management of this illness. We hope that our case sheds further light on the existing knowledge of this syndrome.

## Introduction

Laurence Moon syndrome and Bardet Biedl syndrome are rare genetic ailments with some overlapping characteristics. Both are considered to be distinct syndromes because they exhibit mutations in different genes. Recent studies suggest that Laurence Moon syndrome involves a genetic defect in the PNPLA6 gene [1]. In contrast, Bardet Biedl syndrome is associated with a mutation in the BSS gene, which encodes BSS proteins. These proteins are essential for the normal functioning of organs. There has been a long-standing confusion regarding these syndromes because there is a probability of the BSS gene mutation being an underlying occurrence in both. Clinically, Laurence Moon syndrome (LMS) is usually associated with spasticity and the absence of polydactyly [2]. Laurence Moon Bardet Biedl syndrome, however, is a rare autosomal recessive genetic disorder that presents with a varied range of phenotypes. Clinical features include decreased vision, developmental delay, obesity, moon face, and polydactyly. These dysfunctions critically impact the mental faculties and personal life of a patient. The affected individuals are usually a product of consanguineous marriages [3]. In most instances, a diagnosis is made in early childhood, but on rare occasions, it can be delayed. The factors typically responsible for this delay are a lack of awareness and knowledge associated with this disease. Acute onset of weight loss, increased thirst, and frequent urination pointing toward diabetes mellitus may be the only presenting complaint in some patients. A detailed history might reveal poor academic performance and delayed puberty along with decreased vision [4]. The authors present a case report of a young female with Laurence Moon Bardet Biedl syndrome, who recently got admitted to the medicine ward of FaujI Foundation Hospital (FFH), Islamabad, due to suspected gastroenteritis.

## Case presentation

A 29-year-old female, a diagnosed case of Laurence Moon Bardet Biedl syndrome since age 10, presented to the medicine outpatient department (OPD) of FFH with a complaint of an undocumented and high-grade fever for the past four days, which subsided on taking acetaminophen. The fever was associated with rigor and chills, as well as a single episode of vomiting in the past 24 hours. She has been known to suffer from co-morbidities such as diabetes mellitus and hypertension since the age of 10. There was no history of hematemesis, diarrhea, or any urinary problems, but complaints of a decreased appetite and occasional nausea were reported. The patient’s diabetes mellitus had always been uncontrolled despite being on insulin for the past 19 years. She was also on anti-hypertensive medication for nearly two decades. Family history revealed that the patient was a product of a consanguineous marriage.

**Figure 1:**
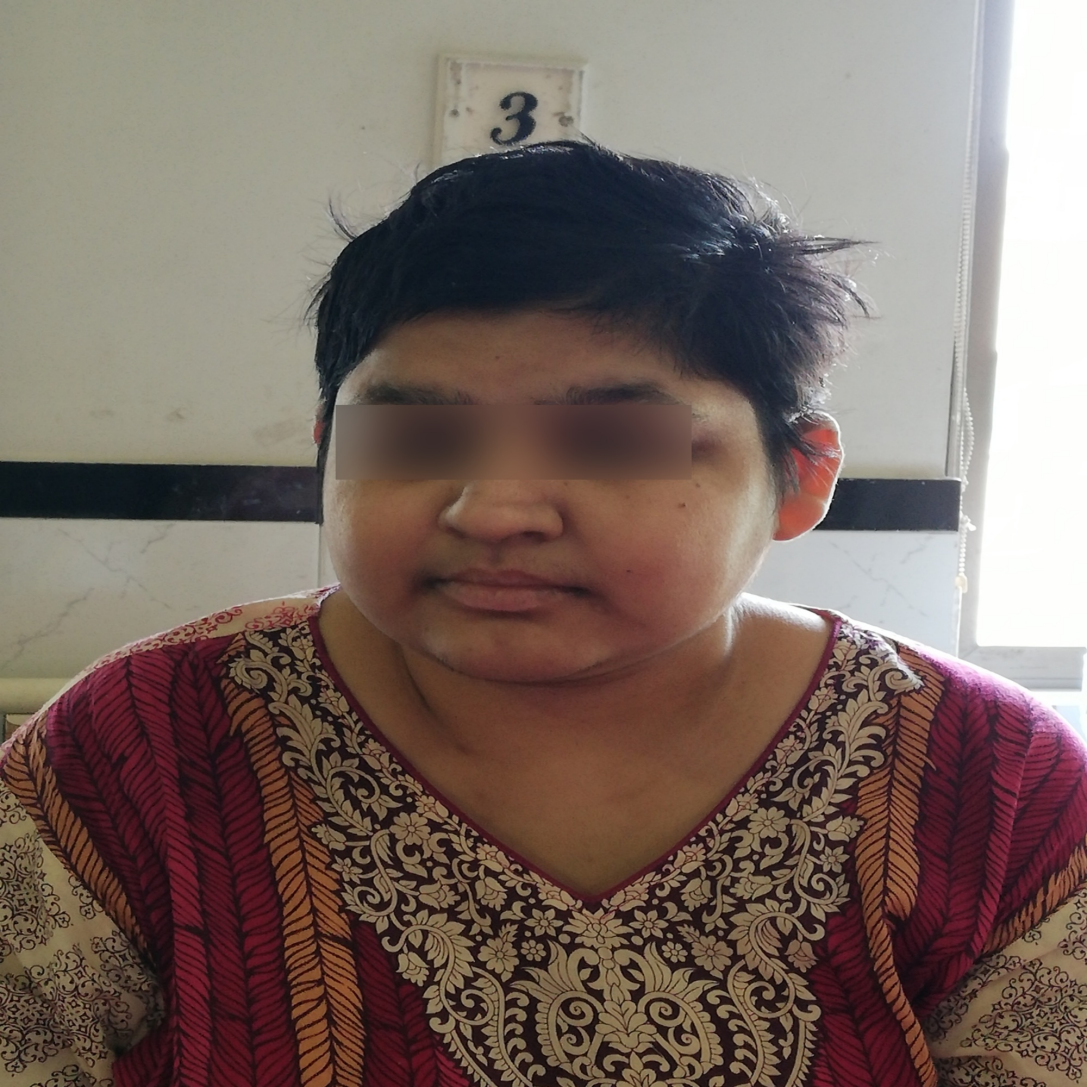
Moon-like face of patient with Laurence Moon Bardet Biedl syndrome

**Figure 2:**
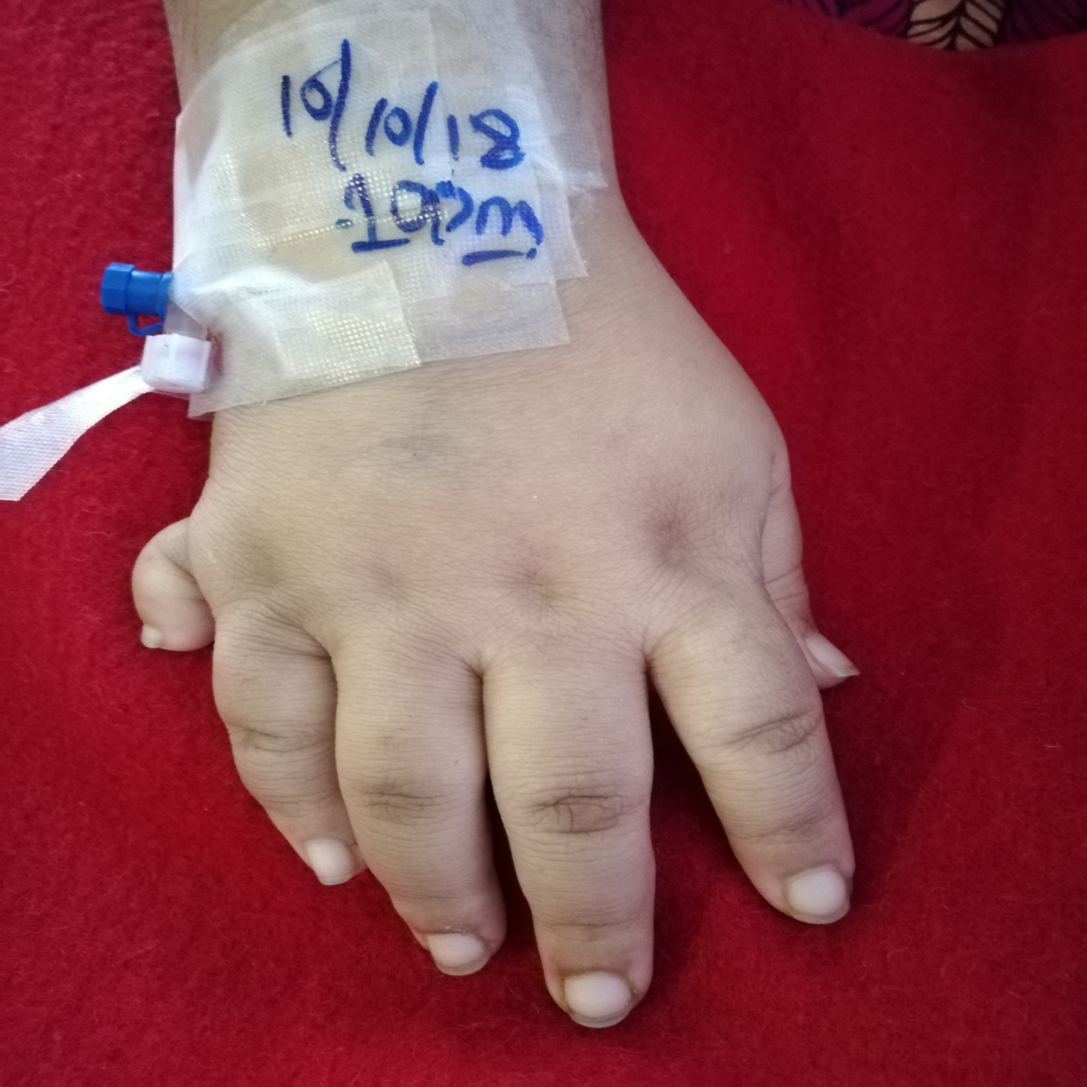
Polydactyly of right hand

**Figure 3:**
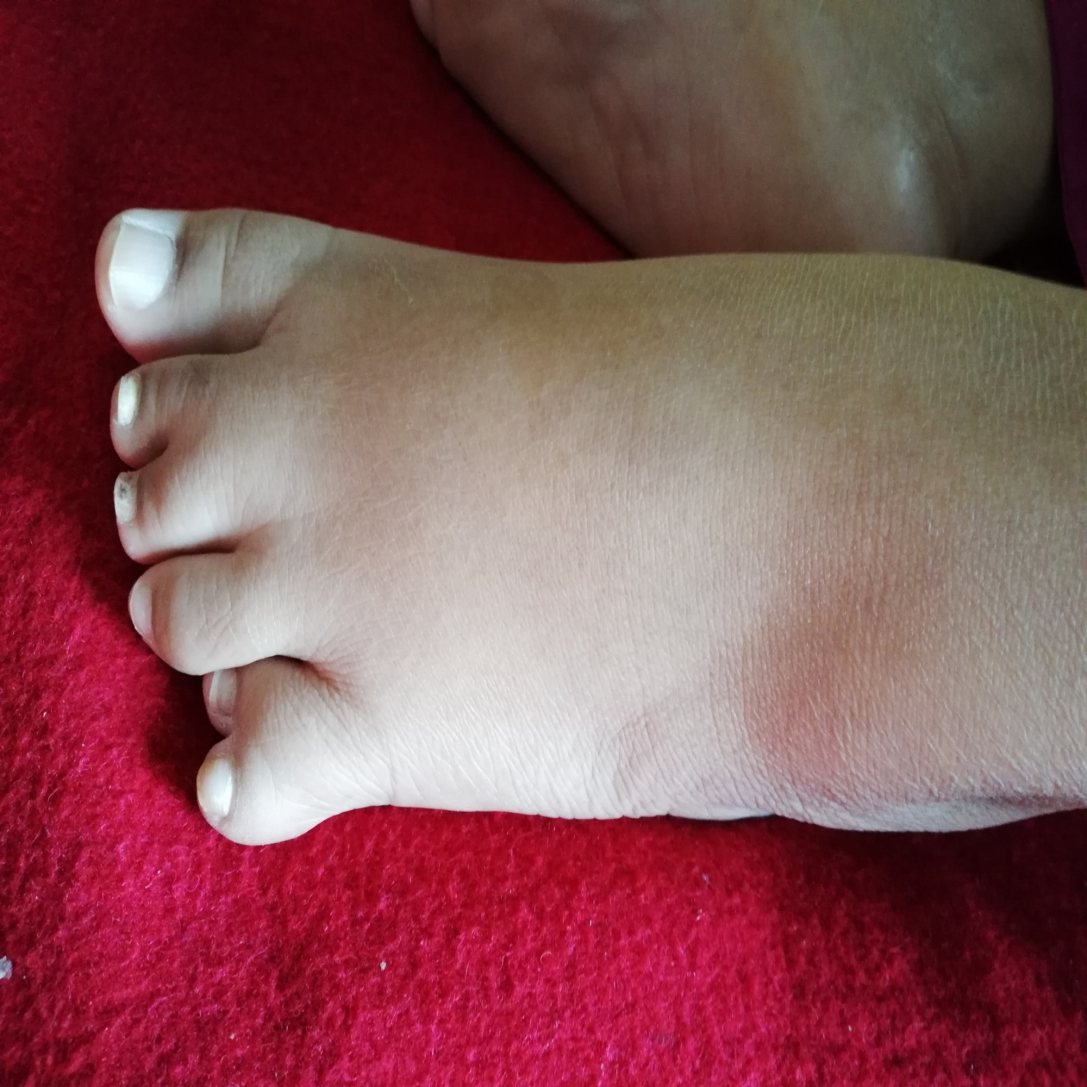
Polydactyly of left foot

At the time of initial presentation to the hospital, her vital signs were: blood pressure of 150/85 mmHg, heart rate of 75 beats per minute, oxygen saturation of 94% on room air, respiratory rate of 25 breaths per minute, and temperature of 101 degrees Fahrenheit. She was in apparent distress. On physical examination, her abdomen was soft and non-tender and heart sounds were normal. Expiratory crepitations were heard on lung auscultation, due to which a chest X-ray was ordered. On skin examination, there were patchy areas of thickened and darkened skin, reflecting acanthosis nigricans, an indicator of insulin resistance. On visual examination, visual acuity was considerably decreased due to retinitis pigmentosa. The patient was markedly obese, her body mass index (BMI) was calculated to be 33 kg/m2 and she had a characteristic moon-like face (Figure 1). She also had an extra digit on her right hand and left foot, indicating polydactyly (Figures 2-3). According to her attendant, she had no regular check-ups and visited the local general practitioner (GP) or hospital only when she got severely sick. They declined any follow-up dates given by health care professionals.

A number of laboratory investigations were carried out; the investigations and their results are shown in Table 1.

**Table 1:** Lab investigations and results WBC: white blood cell; MCV: mean corpuscular volume; MCHC: mean corpuscular hemoglobin concentration; PT: prothrombin time; APTT: activated partial thromboplastin time; INR: international normalized ratio

Investigations	Results (Normal Values)
WBC	14 (5-10 cells/mcl)
Hemoglobin	6.5 (12-14g/dl)
Hematocrit	23.1 (36-42%)
MCV	60 (80-95fl)
MCH	16.8 (27-32pg)
MCHC	28.1 (31.5-34.5g/dl)
Platelets	278 (100-400 mm^3^)
Urea	6.8 (3.3-8.3mmol/l)
Creatinine	0.8 (0.7-1.2mg/dl)
Blood Glucose	14.6 (<11.1mmol/l)
Sodium	141 (136-141mmol/l)
Potassium	3.9 (3.4-5mmol/l)
Chloride	98 (98-106mmol/l)
PT	14 seconds (12-14 seconds)
APTT	30 seconds (30-34 seconds)
INR	1 (up to 1.5)

Urine analysis was carried out, which revealed no positive findings. Due to her low hemoglobin, tests for serum iron, B12, ferritin, and reticulocyte count were also carried out, all of which came out to be normal. Only iron came out low, thereby indicating iron deficiency anemia. Due to the threat of cardiac problems in patients with Laurence Moon Bardet Biedl syndrome and due to high blood pressure, electrocardiography (ECG) and echocardiography were ordered, which came out normal. The patient was started on a number of medications, which included acetaminophen for fever, insulin to control her blood glucose, angiotensin-converting enzyme (ACE) inhibitor to control her blood pressure. Aspirin was also given, as well as iron sucrose injection for her iron deficiency anemia. Acute febrile illness (gastroenteritis) was diagnosed, and the patient was started on levofloxacin. On the third day of hospital admission, the patient became afebrile and was discharged the next day. Her attendants were advised to be vigilant in maintaining a normal blood glucose level and blood pressure through regular exercise and medications.

## Discussion

The first known case of this disease was reported by Laurence and Moon in 1886. Another syndrome with comparable findings was proposed by Bardet Biedl in the year 1920. Due to the similarity in the two conditions, it was established that Bardet Biedl Syndrome and Laurence Moon Syndrome are different forms of the same ailment. The universally accepted term to describe the illness thus became Laurence Moon Bardet Biedl syndrome (LMBBS), a name put forth by Solis-Cohen and Weiss [5]. The syndrome has always spread confusion in medical literature because of its dual disease components and lack of distinguishing features. Some researchers are even convinced that Bardet Biedl is actually a subdivision of Laurence Moon syndrome. The dominating features of Laurence Moon syndrome are progressive spastic paraparesis, retinal pigmentary degeneration, mental retardation, and hypogonadism. Polydactyly and obesity, however, are usually distinctive features of Bardet Biedl syndrome [6].

LMBBS is a rare autosomal recessive condition, with very few recorded cases. It can be characterized by structural and functional abnormalities of organs and tissues. In recent studies, the number of genes associated with this disease has been reported to be as many as 14. These are called BBS genes, which are responsible for encoding BBS proteins. Some of the genes associated include BBS1, BBS2, ARL6/BBS3, BBS4, BBS5, MKKS/BBS6, BBS7, TTC8/BBS8, B1/BBS9, BBS10, and TR1M32/BBS11 [7]. The functioning of cell structures, such as cell cilia, is dependent on the proteins formed by these genes. Any change in these genes directly influences the functioning of cilia, disturbing the chemical signaling pathways that operate throughout the process of development. This leads to a decline in sensual perception. In this way, gene mutations are responsible for ciliopathies leading to the disease [8].

The prevalence of genetic disorders, such as LMBBS, differs in different populations. There is very little literature regarding this disease due to its rarity. Developed countries generally have more accurate prevalence rates as compared to third-world countries like Pakistan. In North America and Europe, the prevalence rates of LMBBS are 1:140000 and 1:160000, respectively. In geographically isolated populations and places with consanguineous marriage trends, the rate of occurrence is believed to be much greater [6]. Consanguinity is the social norm in many Middle Eastern countries, such as Kuwait, Saudi Arabia, Iran, as well as Pakistan. It has been proven to be a major attributing factor to disease incidence. In families from these parts of the world, about 10% of the total genome has been found to be of a homozygous nature. The pattern of inheritance is usually that a single ancestor passes the gene on to the parents of the diseased child. When the child is homozygous, i.e., he carries mutations in both alleles of a gene, he exhibits symptoms of illness, unlike his parents who are just carriers. In a country like Pakistan, around 60% of all marriages are consanguineous in nature. What’s even more troubling is that out of these marriages, 80% tend to be among first cousins, thereby increasing the risk of homozygous mutations. Despite this alarming reality, the prevalence of LMBBS is still unknown in Pakistan, and a large number of cases go undiagnosed. Up until now, there have only been nine reports of 18 families with these mutations [9].

Patients suffering from LMBBS experience an onset of symptoms within the first decade of their life. The first complaint is often poor vision at night, with nystagmus being a common finding [5]. The features of the disease can be classified into primary and secondary type. Primary features include polydactyly, obesity, learning disabilities, rod-cone dystrophy, renal abnormalities, and hypogonadism. Secondary features can be a developmental delay, brachydactyly, speech disturbance, ataxia, diabetes mellitus, spasticity, hearing loss, left ventricular hypertrophy, and polyuria/polydipsia. For the purpose of diagnosis, a patient must meet a set criterion. They must present with four primary features or three primary and two secondary features [10]. Our patient was a known diabetic, hypertensive, obese, with obvious vision loss, as well as polydactyly of both hands and feet. Learning disabilities and developmental delay were also present. Learning disability may be attributed to mental retardation and decreased visual acuity.

Due to a lack of existing knowledge, there is no cure for this disease to date. The only relief that can be offered is symptomatic management, which requires a multidisciplinary approach targeted at each individual presenting feature. Regular monitoring of the lipid, glucose, renal, and endocrine systems is paramount. Maintenance of weight and blood pressure, as well as regular eye examinations, are necessary for making sure the disease stays under control. A regular check-up for any degenerative eye changes is of paramount importance, as further deterioration can be prevented by early diagnosis. Similarly, the patient must also be screened for complications of diabetes and hypertension. The patient and their caretakers should be educated in regards to the disease and its symptomatic management. Visual aids, speech and behavior therapy, as well as schools for disadvantaged children are all available for those seeking help. Cosmetic surgery for the removal of extra digits and hormone replacement therapy are also viable options.

## Conclusions

The rarity of LMBBS has resulted in a severe lack of awareness regarding the incidence of disease in the Pakistani population. The general populace must be educated on the dangers of consanguineous marriages; genetic counseling would go a long way in decreasing the incidence of the disease. Health care professionals must be encouraged to know more about the disease, its risk factors, as well as its management. Efforts made towards this will considerably control the disease and limit unwarranted complications. The quality of life of affected individuals can be significantly improved by early diagnosis and proper follow-up, which is necessary to ensure that appropriate care is being given to the patient. We believe better and more efficient methods of diagnosis and management can only be introduced when the doctors and public alike are equipped with an appropriate amount of knowledge relating to the illness.
